# CAN GOLF INFLUENCE GAIT AND COGNITION IN OLDER ADULTS?

**DOI:** 10.1093/geroni/igz038.601

**Published:** 2019-11-08

**Authors:** Nicole Marcione, Andrea Du Bois, Steven Castle, George Salem

**Affiliations:** 1 University of Southern California, Los Angeles, California, United States; 2 University of California, Los Angeles, Los Angeles, California, United States

## Abstract

Gait speed and cognition are important predictors of successful aging. Both slow gait speeds and cognitive decline are associated with poor health outcomes, including hospitalization, falls, institutionalization and death. Exercise interventions can improve both gait and cognitive performance in older adults. Golf is a multimodal, cognitively-challenging physical activity. The purpose of the present study was to examine the influence of a 12-week golf intervention on walking performance and cognition in older adults. Gait speed and cognition were measured in intervention (INT) and control (CON) groups (n=20) before and after a 12-week period. The INT participated in a 12-week golf training program (2 x weekly; 90 min per session). All participants completed six-minute walk test (6MWT), fast single-task gait speed (STGS), fast dual-task gait speed (DTGS) with a subtraction by 3s task, California Verbal Learning Test (CVLT) and National Institute Health Toolbox-Cognition (NIH-C). 2x2 (Time*Group) Mixed ANOVA revealed significant time*group interactions for 6MWT (p=0.05), STGS (p=0.02), DTGS (p=0.01), CVLT (p=0.01), and NIH-C Fluid Cognition (p=0.06) had a trend towards significance. Post-hoc t-tests demonstrated that INT significantly improved their pre-to-post 6MWT, STGS, DTGS, CVLT, and NIH-C Fluid. CON had no significant pre-to-post intervention changes. Participants in the 12-week golf training program improved gait and cognitive performance, compared to CON. These results provide evidence that golf, as a cognitively-challenging physical activity, may improve physical and cognitive function, leading to attenuated risk for poor health outcomes, maintaining independence and improved quality of life.

